# Work-related distress and professional quality of life in a medical-school workforce: a primary-care occupational-health perspective

**DOI:** 10.3389/fmed.2026.1933465

**Published:** 2026-09-03

**Authors:** Falah Mohammed Almarzooqi, Saif Al-Shamsi, Syed Fahad Javaid, Afaf Alblooshi, Faten AlRadini

**Affiliations:** 1 Department of Family Medicine, College of Medicine and Health Sciences, United Arab Emirates University, Al Ain, United Arab Emirates; 2 Department of Internal Medicine, College of Medicine and Health Sciences, United Arab Emirates University, Al Ain, United Arab Emirates; 3 Department of Psychiatry, College of Medicine and Health Sciences, United Arab Emirates University, Al Ain, United Arab Emirates; 4 Department of Medical Education, College of Medicine and Health Sciences, United Arab Emirates University, Al Ain, United Arab Emirates; 5 Department of Family and Community Medicine, College of Medicine, Princess Nourah bint Abdulrahman University, Riyadh, Saudi Arabia

**Keywords:** burnout, compassion satisfaction, family medicine, occupational health, primary care, professional quality of life, United Arab Emirates, work-related stress

## Abstract

**Background:**

Work-related distress may present in primary care through nonspecific physical, psychological, or functional complaints. This study assessed professional quality of life in a United Arab Emirates medical-school workforce through primary-care and occupational-health perspectives.

**Methods:**

A cross-sectional electronic survey was conducted among employees of the College of Medicine and Health Sciences, United Arab Emirates University, between October 2025 and February 2026. The sampling frame comprised 373 employees. Professional quality of life was assessed using the Professional Quality of Life Scale, Version 5. Descriptive statistics, reliability analysis, subgroup comparisons, correlations, and sensitivity analyses were performed.

**Results:**

Of 373 eligible employees, 162 responded to the survey invitation, 140 were retained after consent confirmation, 109 reached the ProQOL section, and 108 had usable ProQOL subscale scores. Faculty members accounted for 56.9% of respondents and non-faculty employees for 43.1%. Mean scores were 38.88 (SD = 5.70) for compassion satisfaction, 22.35 (SD = 5.13) for burnout, and 23.67 (SD = 6.24) for secondary traumatic stress. Compassion satisfaction was mostly moderate or high, burnout was low or moderate, and secondary traumatic stress was mostly low or moderate. Cronbach’s alpha coefficients were 0.857, 0.747, and 0.838, respectively. No statistically significant differences were detected between faculty and non-faculty employees across ProQOL domains. Compassion satisfaction was strongly and negatively correlated with burnout (*r* = −0.620, *p* < 0.0001), while burnout was moderately and positively correlated with secondary traumatic stress (*r* = 0.535, *p* < 0.0001).

**Conclusion:**

Professional meaning was generally preserved, but workload-related strain remained evident. The findings support attention in primary care to occupational context, functioning, recovery, and work-life boundaries. They also support coordinated consideration of workload and access to support by institutional leadership, human resources, and occupational-health services. These implications are hypothesis-generating because specific job demands, resources, and intervention effects were not measured.

## 1 Introduction

Burnout is an occupational phenomenon resulting from chronic workplace stress that has not been successfully managed (1). It is commonly described through exhaustion, mental distance or cynicism, and reduced professional efficacy, although definitions and measurement approaches vary across studies (2, 3). Burnout alone, however, does not capture the full professional experience of working adults. The Professional Quality of Life Scale, Version 5 (ProQOL-5), assesses three related but distinct domains: compassion satisfaction, burnout, and secondary traumatic stress (4). This distinction is clinically useful because professional meaning and occupational strain may coexist.

Work is an important determinant of health, functioning, prevention needs, social participation, and help-seeking. Employment conditions are recognized as social determinants of health, and long working hours have been linked with substantial cardiovascular disease burden (5, 6). Recent work-health literature further emphasizes that mental health at work is shaped by interacting organizational, psychosocial, and individual factors, and that workplace mental-health strategies should address working conditions as well as individual support (7–9). Work-related distress may present in primary care through fatigue, sleep disturbance, anxiety, low mood, headaches, musculoskeletal symptoms, impaired functioning, recurrent consultations, sickness absence, or deterioration in chronic disease control rather than as an explicit occupational complaint (10–14).

This occupational-health perspective is directly relevant to family medicine. Primary care is organized around first-contact access, continuity, comprehensiveness, coordination, and whole-person care (15, 16). Family physicians are therefore well positioned to identify occupational context when working adults present with nonspecific physical, psychological, or functional complaints (17–19). Workforce wellbeing studies can contribute to family medicine by identifying early patterns of strain in defined working-adult populations, even when they do not directly measure healthcare utilization, sickness absence, or clinical diagnoses.

A medical-school workforce is a useful occupational community for examining professional quality of life because it includes diverse educational, administrative, technical, research-support, student-facing, clinical, and institutional service roles (18, 20–23). Recent higher-education literature shows that psychological distress, job insecurity, burnout, and intervention needs remain relevant among university staff and faculty, including in post-pandemic academic settings (24–28). Although only some employees have direct clinical responsibilities, many contribute to helping, service, mentoring, student support, or institutional functions (4, 20, 22, 24). Faculty and non-faculty employees may differ in autonomy, workload patterns, academic expectations, operational duties, student contact, and institutional recognition, but both groups share the same organizational environment (21–23). A whole-workforce approach is therefore important because occupational strain should not be assumed to affect only clinicians, academics, or high-status professional groups.

The Job Demands–Resources model provides an organizational framework for interpreting professional quality of life. The model proposes that sustained job demands, such as workload, emotional demands, role ambiguity, and after-hours work, may consume employees’ physical and psychological resources, whereas job resources, such as autonomy, supervisory support, role clarity, recognition, and opportunities for recovery, may support motivation and wellbeing. Within this framework, professional meaning and occupational strain may coexist when meaningful aspects of work remain present despite substantial demands. In the present study, the ProQOL-5 was used to measure compassion satisfaction, burnout, and secondary traumatic stress, whereas the Job Demands–Resources model was used only as an interpretive framework. Specific job demands and job resources were not directly measured; therefore, the study did not formally test the model or its causal pathways (29).

This study aimed to assess compassion satisfaction, burnout, and secondary traumatic stress among faculty and non-faculty employees in a UAE medical-school workforce and to interpret these patterns through a family medicine and occupational-health lens. Based on prior and recent ProQOL, higher-education, academic-medicine, and healthcare workforce wellbeing literature, we hypothesized that compassion satisfaction would be generally preserved, that burnout would be inversely associated with compassion satisfaction and positively associated with secondary traumatic stress, and that faculty/non-faculty differences would be smaller than expected because both groups share the same institutional environment (21–23, 26, 30–39). The study does not establish clinical diagnoses, causality, healthcare utilization, sickness absence, or occupational-health referral patterns; rather, it describes professional quality-of-life patterns in a defined working-adult population. The Job Demands–Resources model guided the organizational interpretation of the findings but was not empirically tested because specific job demands and resources were not measured.

Although ProQOL and burnout have been examined in healthcare, academic, and higher-education settings, prior work has commonly focused on clinicians, academic physicians, faculty, nurses, or broad university employee groups rather than the full medical-school workforce within a UAE academic medical institution (4, 20–26, 30–39). To our knowledge, few studies have assessed compassion satisfaction, burnout, and secondary traumatic stress across both faculty and non-faculty employees in this setting. This study addresses that gap by examining a defined UAE medical-school workforce and interpreting the findings through a family medicine and occupational-health lens, highlighting the relevance of professional quality of life for primary-care recognition of work-related distress, functioning, recovery, and help-seeking among working adults (7–9, 17–19).

## 2 Materials and methods

### 2.1 Study design and setting

This cross-sectional survey assessed professional quality of life among employees at the College of Medicine and Health Sciences (CMHS), United Arab Emirates University (UAEU). Data were collected between October 2025 and February 2026 using a self-administered electronic questionnaire hosted on Research Electronic Data Capture (REDCap) at the UAEU. REDCap is a secure, web-based software platform designed to support data capture for research studies through an intuitive interface for validated data entry, audit trails for tracking data manipulation and export procedures, automated export to common statistical packages, and procedures for data integration and interoperability with external sources (40, 41).

The study was designed as a cross-sectional occupational-health-informed assessment of a defined working-adult population rather than as a clinical diagnostic survey. It did not assess presenting symptoms, primary-care consultations, sickness absence, chronic disease control, occupational-health referral, counseling use, return-to-work outcomes, or clinical diagnoses. Its primary-care relevance lies in examining work-related professional quality-of-life domains that may inform clinical assessment of occupational context, functioning, recovery, prevention needs, and help-seeking among working adults (17–19). CMHS includes academic, administrative, technical, teaching, research, and service-related roles, making it an appropriate setting for examining professional quality of life across a medical-school workforce rather than among academic faculty alone.

### 2.2 Participants and recruitment

The target population included all adults employed at CMHS during the study period. At the time of the survey, CMHS had 373 employees, who constituted the intended institutional sampling frame. Faculty members were defined as academic employees holding the rank of Assistant Professor, Associate Professor, or Professor. Non-faculty employees included employees who did not hold these academic faculty ranks, including administrative staff, technical staff, instructors, research assistants, and respondents who selected “Other” as their position category.

The term “medical-school workforce” is used throughout the manuscript to emphasize that the analytic population includes employees who contribute to the educational, research, administrative, technical, and service functions of the medical school. This terminology also avoids assuming that occupational wellbeing in a medical school is primarily a faculty issue.

A whole-workforce recruitment strategy was used. The survey invitation was made available to all 373 employees in the institutional sampling frame through institutional email, flyers, and announcements circulated through the Dean’s Office. No random or stratified selection of employees was undertaken. Recruitment therefore constituted a total-population invitation (attempted census), while the achieved sample was a voluntary, self-selected non-probability sample. Participation was voluntary, and the analytic dataset was de-identified before analysis. Among the 373 eligible CMHS employees, 162 responded to the survey invitation, yielding an initial institutional response rate of 43.4%.

### 2.3 Instrument

Professional quality of life was assessed using the ProQOL-5 (Supplementary Figure S1), a 30-item self-report instrument that measures three subscales: compassion satisfaction, burnout, and secondary traumatic stress (4). Each subscale contains 10 items rated on a five-point Likert scale. Compassion satisfaction refers to the positive aspects of helping or service-oriented work, including the sense of meaning, pleasure, competence, and fulfillment derived from one’s professional role. Burnout refers to negative work-related experiences characterized by exhaustion, frustration, reduced perceived effectiveness, and difficulty coping with occupational demands. Secondary traumatic stress refers to stress reactions associated with indirect exposure to others’ distressing or traumatic experiences through professional responsibilities.

Permission to use the ProQOL-5 was obtained, and the instrument was used without modification. Because the ProQOL-5 uses helping and service-oriented language, “helping” was interpreted broadly in this medical-school context to include educational, administrative, technical, student-support, research-support, mentoring, clinical, and institutional service roles. However, the instrument was not adapted to distinguish direct clinical exposure from indirect student-facing, administrative, or service-related exposure. Findings for secondary traumatic stress should therefore be interpreted cautiously.

Subscale scores were calculated according to official ProQOL scoring guidance (4). Each ProQOL subscale includes 10 items, and burnout scoring includes reverse scoring for specified items. A subscale score was considered usable when at least 8 of the 10 items for that subscale were available. If 8 or 9 items were answered, the mean of the answered items was multiplied by 10 to create the subscale score. Participants without any usable ProQOL subscale score were excluded from subscale analyses. The three subscales were scored separately and were not combined into a total score. Scores were interpreted descriptively using the official ProQOL-5 bands: 22 or less was categorized as low, 23–41 as moderate, and 42 or more as high (4). These categories were used to summarize score distributions and were not interpreted as diagnostic thresholds.

### 2.4 Data management and statistical analysis

After survey closure, data were exported from REDCap using its standard export procedures. Records were retained for analysis only when explicit consent was documented. For REDCap repeated-instrument exports, main survey rows and ProQOL rows were linked using the study record ID. When more than one ProQOL row was available for a record, the row with the largest number of answered ProQOL items was retained. Data were then screened for completeness, missing values, and response inconsistencies before analysis.

Categorical variables were summarized as frequencies and percentages. Continuous variables were summarized using means and standard deviations. Medians and interquartile ranges were also reported for the ProQOL subscale scores. The distributions of the ProQOL subscale scores were assessed using histograms and normal Q-Q plots, with attention to marked skewness and outlying observations. Because the study was exploratory and cross-sectional, analyses were interpreted descriptively. Non-significant subgroup comparisons were not treated as evidence of equivalence, particularly where subgroup sizes were small. Effect sizes and detectable-effect analyses were included to support cautious interpretation of null findings.

For item-level descriptive analysis, high endorsement was defined as an interpreted item value of 4 or 5. For compassion satisfaction and secondary traumatic stress items, this reflected the original response direction. For burnout items requiring reverse scoring, responses were reversed before item-level interpretation, so higher interpreted values reflected greater burnout burden. Item-level findings were used descriptively only and were not used as diagnostic thresholds.

The primary outcomes were the three ProQOL subscale scores: compassion satisfaction, burnout, and secondary traumatic stress. Internal consistency reliability was assessed for each ProQOL subscale using Cronbach’s alpha; alpha coefficients of ≥0.70 were considered acceptable for exploratory analysis (42). Subgroup comparisons were conducted by faculty/non-faculty category, gender, UAEU tenure group, and work environment. The UAEU tenure comparison used 0–3 years versus more than 6 years; the 4–6 year category was retained descriptively and was not included in this binary comparison. Comparisons were completed only when at least two groups had five or more respondents with usable data. Because only one respondent reported remote work, work-environment comparisons were limited to office-based versus hybrid respondents and were interpreted descriptively. Welch two-sample t-tests were used for two-group comparisons. Mann–Whitney U tests were used as a sensitivity analysis for these comparisons. Pearson correlations were used to examine associations among ProQOL subscales using pairwise complete observations. Detectable-effect analyses were conducted to support the interpretation of non-significant subgroup comparisons. No adjustment was made for multiple comparisons. All analyses were conducted using R statistical software (R Foundation for Statistical Computing, Vienna, Austria) (43). A two-sided *p*-value of <0.05 was considered statistically significant.

## 3 Results

### 3.1 Participant flow and characteristics

Of 373 eligible CMHS employees, 162 responded to the survey invitation, yielding an initial institutional response rate of 43.4%. After records without explicit consent were excluded, 140 records were retained, corresponding to 37.5% of the eligible workforce. Of these, 109 participants reached the ProQOL section and provided at least one ProQOL item response. Complete 30-item ProQOL data were available for 107 participants, corresponding to 28.7% of the eligible workforce. One additional partial ProQOL record was scoreable at the subscale level using the prorating rule, giving 108 participants with at least one usable ProQOL subscale score, corresponding to 29.0% of the eligible workforce (Table 1).

**Table 1:** Participant flow and ProQOL analytic sample.

Stage	*n* (%)
Eligible CMHS employees	373 (100.0)
Survey responses exported	162 (43.4)
Consent-confirmed records	140 (37.5)
Reached ProQOL section	109 (29.2)
Usable ProQOL subscale scores	108 (29.0)
Complete 30-item ProQOL data	107 (28.7)

Percentages were calculated using the eligible CMHS workforce as the denominator (*N* = 373).

Among respondents with at least one ProQOL item response (*n* = 109), faculty members accounted for 56.9% and non-faculty employees for 43.1%. Females represented 56.0% of respondents and males 44.0%. Most participants were aged 35–44 years (40.4%) or 45–54 years (30.3%). Most were full-time employees (94.5%), had worked at UAEU for more than 6 years (67.9%), and reported an office-based work environment (88.1%) (Supplementary Table S1).

### 3.2 ProQOL subscale scores and categories

Subscale summaries were calculated among participants with usable scores for each subscale, with 108 usable scores for compassion satisfaction, burnout, and secondary traumatic stress. Burnout and secondary traumatic stress each included one prorated subscale score, while compassion satisfaction did not include any prorated scores.

Mean compassion satisfaction was 38.88 (SD = 5.70), mean burnout was 22.35 (SD = 5.13), and mean secondary traumatic stress was 23.67 (SD = 6.24). Compassion satisfaction was mostly moderate or high: 74 participants (68.5%) were in the moderate category and 33 (30.6%) were in the high category. Burnout was divided between low and moderate categories, with no respondents categorized as high. Secondary traumatic stress was mostly low or moderate, with 2 participants (1.9%) categorized as high (Table 2). Graphical assessment showed that compassion satisfaction and burnout were approximately symmetric, while secondary traumatic stress showed some right-skew.

**Table 2:** ProQOL subscale scores and descriptive categories.

Subscale	Mean (SD)	Median (IQR)	95% CI	Low *n* (%)	Moderate *n* (%)	High *n* (%)
Compassion satisfaction	38.88 (5.70)	39.00 (35.75–42.25)	37.79–39.97	1 (0.9)	74 (68.5)	33 (30.6)
Burnout	22.35 (5.13)	22.00 (19.00–25.25)	21.37–23.33	56 (51.9)	52 (48.1)	0 (0.0)
Secondary traumatic stress	23.67 (6.24)	23.38 (19.00–27.00)	22.48–24.86	46 (42.6)	60 (55.6)	2 (1.9)

CI, confidence interval; IQR, interquartile range; SD, standard deviation. Descriptive categories use ProQOL interpretation bands.

Internal consistency was acceptable to good across subscales, with 108 usable records for each reliability estimate. Cronbach’s alpha was 0.857 for compassion satisfaction, 0.747 for burnout, and 0.838 for secondary traumatic stress (Table 3).

**Table 3:** Internal consistency reliability of ProQOL subscales.

Subscale	Usable n	Items	Cronbach’s alpha	Standardized alpha	Average inter-item r
Compassion satisfaction	108	10	0.857	0.857	0.375
Burnout	108	10	0.747	0.752	0.232
Secondary traumatic stress	108	10	0.838	0.842	0.347

### 3.3 Item-level findings

Item-level analysis supported the subscale findings. The strongest compassion satisfaction endorsement was for getting satisfaction from being able to help people (89.9% high endorsement), followed by being happy about choosing this work (77.8%), feeling proud of what one can do to help (76.9%), and feeling satisfied with work (75.0%). The strongest burnout-related item was feeling overwhelmed because workload seemed endless (33.3% high endorsement), followed by feeling bogged down by the system (23.1%). For secondary traumatic stress, the most strongly endorsed item was preoccupation with more than one person helped (59.6%), followed by difficulty separating personal life from work as a helper (22.9%).

### 3.4 Subgroup comparisons and correlations

No statistically significant differences were detected between faculty and non-faculty employees in compassion satisfaction, burnout, or secondary traumatic stress. No statistically significant differences were found by gender or by office-based versus hybrid work environment. Participants with 0–3 years of UAEU experience had lower mean compassion satisfaction than those with more than 6 years of UAEU experience, but this difference did not reach conventional statistical significance (*p* = 0.0540). These subgroup findings should be interpreted cautiously because the study was powered mainly to detect moderate differences. Full exploratory subgroup comparisons, including *p*-values and effect estimates, are provided in Supplementary Table S2. The Mann–Whitney U sensitivity analyses did not change the interpretation of the subgroup comparisons.

All three correlations used 108 pairwise complete observations. Compassion satisfaction was strongly and negatively correlated with burnout (*r* = −0.620, *p* < 0.0001). Burnout was moderately and positively correlated with secondary traumatic stress (*r* = 0.535, *p* < 0.0001). Compassion satisfaction was not significantly correlated with secondary traumatic stress (*r* = −0.096, *p* = 0.3232). The correlation sensitivity analysis showed that the smallest detectable correlation at about 80% power was *r* = 0.266. For faculty/non-faculty comparisons, the smallest detectable effect at about 80% power was d = 0.549. Smaller subgroup differences may therefore have gone undetected.

## 4 Discussion

This study examined professional quality of life in a defined UAE medical-school workforce and interpreted the findings through a family medicine and occupational-health lens. The main finding was a mixed profile: professional meaning was generally preserved, while workload-related strain was measurable. Compassion satisfaction was mostly moderate or high, burnout was low or moderate, and secondary traumatic stress was mostly low or moderate. This pattern suggests that employees may remain engaged with meaningful work while still experiencing occupational strain.

The findings should not be interpreted as absence of risk. Almost half of respondents were in the moderate burnout category, and the most strongly endorsed burnout item was feeling overwhelmed because workload seemed endless. This indicates an early-strain profile rather than severe burnout. For primary care, this is clinically relevant because work-related distress may present through fatigue, poor sleep, pain, anxiety, reduced concentration, impaired recovery, sickness absence, or reduced functioning before severe psychological morbidity becomes apparent (10, 12–14, 18, 44). Because clinical outcomes were not measured, this interpretation should be considered hypothesis-generating rather than evidence of direct effects on healthcare utilization or patient outcomes.

The coexistence of preserved compassion satisfaction with measurable burnout is important. It suggests that professional meaning and occupational strain are not mutually exclusive (30–35). A working adult may remain committed to work while also experiencing workload pressure, emotional spillover, impaired recovery, or functional decline. In clinical practice, this means that assessment should not rely only on whether the patient still finds work meaningful; it should also explore workload, recovery, work-life boundaries, perceived support, and whether symptoms interfere with daily functioning or work participation (12, 13, 18, 19, 44). Viewed through the Job Demands–Resources model, the item-level findings concerning an apparently endless workload and feeling bogged down by the system may represent job-demand pressures, whereas preserved compassion satisfaction may indicate that professional meaning and some motivational resources remain present. This provides a possible explanation for the coexistence of relatively high compassion satisfaction with measurable burnout. However, this interpretation remains provisional because the survey did not directly measure workload intensity, autonomy, role clarity, supervisory support, recognition, staffing, or recovery opportunities. The present findings therefore support a JD–R-informed interpretation but do not establish the organizational mechanisms responsible for the observed ProQOL pattern (29).

The absence of statistically significant faculty/non-faculty differences supports a whole-workforce view of occupational context, but it should not be interpreted as evidence of equivalence. The study was powered mainly to detect moderate differences, and the non-faculty category combined heterogeneous roles, including administrative, technical, instructor, research-assistant, and “Other” positions. Faculty and non-faculty employees may have different exposures and resources, but both groups operate within the same institutional ecosystem. Larger studies should examine specific occupational subgroups while preserving the whole-workforce perspective.

### 4.1 Implications for family medicine, primary care, and occupational health

**Figure 1:**
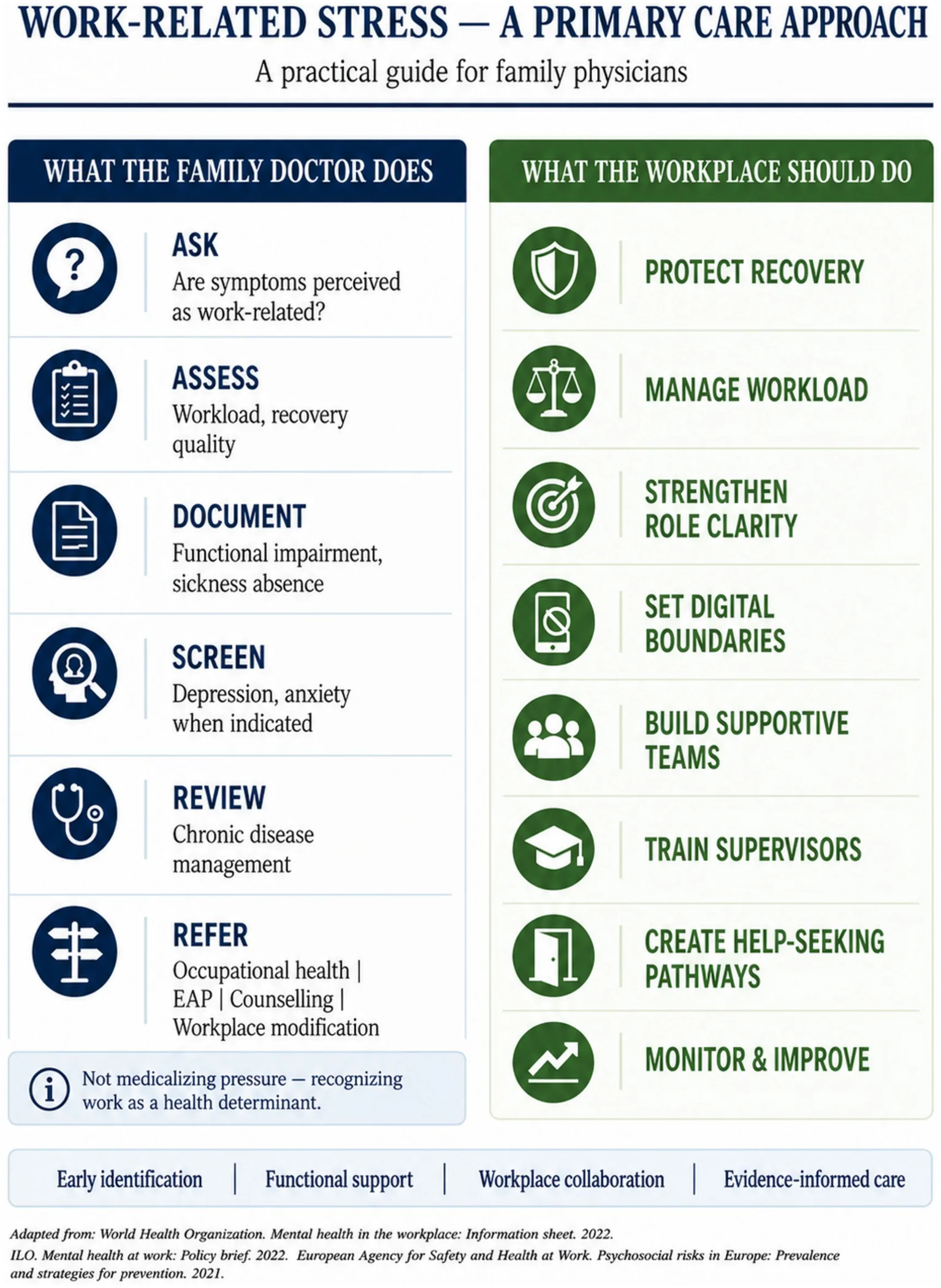
Work-related stress: A primary-care approach. This author-created conceptual synthesis illustrates practical areas for family physicians to consider when assessing working adults with stress-related, sleep-related, mood-related, pain-related, or functional complaints, and shows potential coordination points with workplace and occupational-health systems (7, 13, 17–19, 44–47). The figure was informed by the present findings and published occupational-health guidance; it was not empirically tested as a clinical pathway in this study.

These findings reinforce the importance of asking working adults about occupational context when they present with stress-related, sleep-related, mood-related, pain-related, or functional complaints (17–19). A primary-care approach should avoid medicalizing ordinary work pressure, but it should identify when work conditions contribute to symptoms, impaired functioning, delayed recovery, or worsening chronic disease control (11, 13, 18, 19). Clinically, family physicians can ask whether symptoms are perceived as work-related, assess workload and recovery, explore sleep, mood, pain, concentration, work functioning, and sickness absence, and coordinate with occupational health, counseling, employee-assistance services, or workplace-support systems when appropriate and with the patient’s consent (7, 17, 18, 45–47), Figure 1.

### 4.2 Occupational-health and workplace coordination

Work-related distress cannot be addressed by clinical care alone; recent occupational-health and healthcare workforce literature emphasizes the importance of organizational conditions, work climate, staffing, management support, and system-level prevention alongside individual support (7–9, 18, 38, 39, 45–47). The item-level findings suggest that workload, recovery, and work-life boundaries warrant attention, but the survey did not directly measure workload intensity, after-hours digital work, telepressure, staffing levels, role clarity, perceived organizational support, or psychological detachment (29, 44, 48, 49). Therefore, workplace prevention strategies should be considered plausible implications rather than direct empirical findings. A broader workplace prevention framework is provided as Supplementary Figure S2.

From an organizational-policy perspective, the findings support joint decision-making among medical-school leadership, human resources, occupational-health services, primary-care stakeholders, and employee representatives. The coexistence of preserved professional meaning with workload-related strain suggests that institutional responses should not rely exclusively on individual-level resilience training, counseling, or stress-management programs. Upstream policy options may include periodic workload and staffing review, clearer role expectations, review of administrative processes, realistic expectations for after-hours communication, protected opportunities for recovery, confidential referral pathways, and routine monitoring of workforce wellbeing across faculty and non-faculty groups. These actions should be interpreted as plausible policy implications informed by the observed findings and the Job Demands–Resources framework, rather than as interventions tested by this cross-sectional study (29).

### 4.3 Strengths and limitations

This study has several strengths. It examined the broader medical-school workforce rather than faculty alone, used a validated ProQOL framework that captures both positive and negative professional experiences, reported reliability estimates, and interpreted workforce wellbeing through a clinical and occupational-health lens.

Several limitations should be acknowledged. The cross-sectional design prevents causal inference, and the study was conducted at one medical school, which may limit generalizability. Although the initial institutional response rate was 43.4%, the ProQOL analytic sample represented 29.0% of the eligible workforce; therefore, nonresponse bias cannot be excluded. Self-reported data may also be affected by reporting bias and social desirability bias.

The study did not measure presenting symptoms, primary-care consultations, sickness absence, chronic disease control, occupational-health referral, counseling use, return-to-work outcomes, or clinical diagnoses. Therefore, the findings cannot establish direct effects on primary-care utilization, patient outcomes, or occupational-health outcomes. The non-faculty category was also heterogeneous, and the sample size limited statistical power for subgroup comparisons. Finally, ProQOL categories are descriptive rather than diagnostic, and the absence of qualitative data limits interpretation of how employees understood workload, support, role expectations, and wellbeing resources.

### 4.4 Future research

Future studies should link professional quality-of-life measures with outcomes relevant to primary care, including fatigue, sleep disturbance, pain, mood symptoms, anxiety, work functioning, sickness absence, chronic disease control, healthcare utilization, occupational-health referral, counseling use, and return-to-work outcomes. Longitudinal, mixed-methods, and intervention studies are needed to clarify how workload, recovery, compassion satisfaction, burnout, secondary traumatic stress, symptoms, functioning, and help-seeking are related, particularly because recent faculty-focused reviews suggest that evidence on effective mental-health interventions in academic settings remains limited and heterogeneous (28).

Consistent with the Job Demands–Resources model (29), future intervention development should distinguish demand-reduction strategies from resource-building strategies. Demand-reduction strategies may include workload review, administrative simplification, clearer student-support boundaries, meeting discipline, and expectations for after-hours communication. Resource-building strategies may include role clarity, supervisor training, employee voice, recognition, peer support, psychological safety, and easier access to primary care, occupational health, and counseling pathways. Outcomes should be assessed separately for compassion satisfaction, burnout, and secondary traumatic stress, as these domains may respond differently to the same intervention.

## 5 Conclusion

Professional quality of life in this UAE medical-school workforce was characterized by preserved compassion satisfaction, low-to-moderate burnout, and low-to-moderate secondary traumatic stress. The findings suggest that professional meaning and workload-related strain can coexist in working adults. For family medicine and primary care, this pattern reinforces the importance of asking about occupational context, workload, recovery, work-life boundaries, functioning, sickness absence, and help-seeking when adults present with stress-related, sleep-related, mood-related, pain-related, or functional complaints. At the institutional level, the findings support coordinated attention by medical-school leadership, human resources, and occupational-health services to workload, role clarity, recovery, work-life boundaries, and access to confidential support across the whole workforce. These recommendations require prospective evaluation because the present study did not test organizational interventions. Future studies should link ProQOL domains with symptoms, healthcare utilization, sickness absence, chronic disease control, occupational-health referral, counseling use, and return-to-work outcomes.

## Data Availability

The original contributions presented in the study are included in the article/Supplementary material, further inquiries can be directed to the corresponding author.

## Supplementary material

SUPPLEMENTARY FIGURE S2 Practical workplace burnout-prevention framework. This framework is a conceptual synthesis of plausible prevention targets and should not be interpreted as a direct empirical finding of the present study.
