# Supplementary material for: Work-related distress and professional quality of life in a medical-school workforce: a primary-care occupational-health perspective
Source: Front Med (Lausanne). 2026 Sep 3;13:1933465. doi: 10.3389/fmed.2026.1933465 (PMC13581826; doi:10.3389/fmed.2026.1933465)
Supplement: Supplementary file 1 [file Supplementary_file_1.docx]

**Supplementary Material**

**Supplementary Table 1:** Descriptive subgroup summaries of ProQOL subscale scores by faculty status, gender, UAEU tenure, and work environment.

| **Characteristic** | **n (%)** |
| --- | --- |
| Age group | |
| 35-44 years | 44 (40.4) |
| 45-54 years | 33 (30.3) |
| 55-64 years | 21 (19.3) |
| 25-34 years | 6 (5.5) |
| 65 years and above | 4 (3.7) |
| Under 25 years | 1 (0.9) |
| Gender | |
| Female | 61 (56.0) |
| Male | 48 (44.0) |
| Faculty status | |
| Faculty members | 62 (56.9) |
| Non-faculty employees | 47 (43.1) |
| Highest qualification | |
| PhD | 48 (44.0) |
| Master's degree | 27 (24.8) |
| Bachelor's degree | 13 (11.9) |
| Other | 10 (9.2) |
| MD | 7 (6.4) |
| MBBS | 4 (3.7) |
| Employment status/type | |
| Full-time | 103 (94.5) |
| Part-time | 3 (2.8) |
| Temporary contract | 3 (2.8) |
| Years at UAEU | |
| More than 6 years | 74 (67.9) |
| 4-6 years | 23 (21.1) |
| 1-3 years | 6 (5.5) |
| Less than 1 year | 6 (5.5) |
| Work environment | |
| Office-based | 96 (88.1) |
| Hybrid | 12 (11.0) |
| Remote | 1 (0.9) |

*Note: Percentages were calculated among respondents with at least one ProQOL item response (n = 109). Percentages may not total 100.0 because of rounding.*

**Supplementary Table 2.** Exploratory subgroup comparisons of ProQOL subscale scores

| **Comparison** | **ProQOL outcome** | **Groups compared** | **Total n** | **Test** | **Mean difference (95% CI)** | **Effect size** | **p-value** |
| --- | --- | --- | --- | --- | --- | --- | --- |
| Faculty versus non-faculty employees | Compassion satisfaction | Faculty: n = 61, mean = 39.28, SD = 5.65; non-faculty: n = 47, mean = 38.36, SD = 5.77 | 108 | Welch two-sample t-test | 0.92 (-1.29 to 3.12) | Cohen's d = 0.161 | 0.4108 |
| Faculty versus non-faculty employees | Burnout | Faculty: n = 61, mean = 22.64, SD = 5.50; non-faculty: n = 47, mean = 21.98, SD = 4.65 | 108 | Welch two-sample t-test | 0.66 (-1.28 to 2.60) | Cohen's d = 0.128 | 0.5006 |
| Faculty versus non-faculty employees | Secondary traumatic stress | Faculty: n = 61, mean = 23.30, SD = 6.67; non-faculty: n = 47, mean = 24.16, SD = 5.67 | 108 | Welch two-sample t-test | -0.86 (-3.23 to 1.49) | Cohen's d = -0.139 | 0.4659 |
| Female versus male | Compassion satisfaction | Female: n = 60, mean = 38.40, SD = 5.59; male: n = 48, mean = 39.48, SD = 5.83 | 108 | Welch two-sample t-test | -1.08 (-3.28 to 1.12) | Cohen's d = -0.189 | 0.3326 |
| Female versus male | Burnout | Female: n = 60, mean = 22.32, SD = 4.74; male: n = 48, mean = 22.40, SD = 5.64 | 108 | Welch two-sample t-test | -0.08 (-2.10 to 1.94) | Cohen's d = -0.015 | 0.9382 |
| Female versus male | Secondary traumatic stress | Female: n = 60, mean = 23.58, SD = 5.79; male: n = 48, mean = 23.79, SD = 6.83 | 108 | Welch two-sample t-test | -0.21 (-2.66 to 2.25) | Cohen's d = -0.032 | 0.8699 |
| UAEU tenure: 0-3 years versus >6 years | Compassion satisfaction | 0-3 years: n = 12, mean = 36.92, SD = 3.92; >6 years: n = 74, mean = 39.59, SD = 5.59 | 86 | Welch two-sample t-test | -2.67 (-5.41 to 0.05) | Cohen's d = -0.496 | 0.0540 |
| UAEU tenure: 0-3 years versus >6 years | Burnout | 0-3 years: n = 12, mean = 23.92, SD = 4.87; >6 years: n = 74, mean = 21.77, SD = 5.15 | 86 | Welch two-sample t-test | 2.15 (-1.11 to 5.40) | Cohen's d = 0.420 | 0.1802 |
| UAEU tenure: 0-3 years versus >6 years | Secondary traumatic stress | 0-3 years: n = 12, mean = 25.17, SD = 8.11; >6 years: n = 74, mean = 23.63, SD = 5.70 | 86 | Welch two-sample t-test | 1.54 (-3.73 to 6.80) | Cohen's d = 0.253 | 0.5393 |
| Work environment: hybrid versus office-based | Compassion satisfaction | Hybrid: n = 12, mean = 38.00, SD = 7.75; office-based: n = 95, mean = 38.93, SD = 5.42 | 107 | Welch two-sample t-test | -0.93 (-5.93 to 4.08) | Cohen's d = -0.162 | 0.6945 |
| Work environment: hybrid versus office-based | Burnout | Hybrid: n = 12, mean = 23.75, SD = 6.24; office-based: n = 95, mean = 22.24, SD = 4.98 | 107 | Welch two-sample t-test | 1.51 (-2.54 to 5.56) | Cohen's d = 0.294 | 0.4352 |
| Work environment: hybrid versus office-based | Secondary traumatic stress | Hybrid: n = 12, mean = 24.42, SD = 9.34; office-based: n = 95, mean = 23.59, SD = 5.84 | 107 | Welch two-sample t-test | 0.83 (-5.18 to 6.84) | Cohen's d = 0.132 | 0.7689 |

**Note.** ProQOL = Professional Quality of Life; SD = standard deviation; CI = confidence interval; UAEU = United Arab Emirates University. Mean differences are presented as the first listed group minus the second listed group. All subgroup comparisons were exploratory, and no correction for multiple comparisons was applied. The 4-6 years UAEU tenure group was excluded from tenure comparisons to contrast early-tenure respondents with those reporting more than 6 years at UAEU. Work-environment comparisons were limited to hybrid versus office-based respondents because only one respondent reported remote work.

**Supplementary Material 1:** Professional Quality of Life Scale, Version 5 (ProQOL-5). The official ProQOL-5 instrument is available through www.ProQOL.org and should be submitted as a separate supplementary file if required by the target journal.
